# Antiplasmodial and Cytotoxic Activities of Extracts of Selected Medicinal Plants Used to Treat Malaria in Embu County, Kenya

**DOI:** 10.1155/2020/8871375

**Published:** 2020-07-07

**Authors:** Bibianne Waiganjo, Gervason Moriasi, Jared Onyancha, Nelson Elias, Francis Muregi

**Affiliations:** ^1^School of Pure and Applied Sciences, Department of Biological Sciences, Mount Kenya University, P.O. Box 342-01000 Thika, Kenya; ^2^School of Medicine, Department of Medical Biochemistry, Mount Kenya University, P.O. Box 342-01000 Thika, Kenya; ^3^School of Pharmacy, Department of Pharmacognosy, Mount Kenya University, P.O. Box 342-01000 Thika, Kenya; ^4^School of Postgraduate Studies and Research, Mount Kenya University, P.O. Box 342-01000 Thika, Kenya

## Abstract

Malaria is a deadly disease caused by a protozoan parasite whose mode of transmission is through a female Anopheles mosquito. It affects persons of all ages; however, pregnant mothers, young children, and the elderly suffer the most due to their dwindled immune state. The currently prescribed antimalarial drugs have been associated with adverse side effects ranging from intolerance to toxicity. Furthermore, the costs associated with conventional approach of managing malaria are arguably high especially for persons living in low-income countries, hence the need for alternative and complementary approaches. Medicinal plants offer a viable alternative because of their few associated side effects, are arguably cheaper, and are easily accessible. Based on the fact that studies involving antimalarial medicinal plants as potential sources of efficacious and cost-effective pharmacotherapies are far between, this research was designed to investigate antiplasmodial and cytotoxic activities of organic and aqueous extracts of selected plants used by Embu traditional medicine practitioners to treat malaria. The studied plants included *Erythrina abyssinica* (stem bark), *Schkuhria pinnata* (whole plant), *Sterculia africana* (stem bark), *Terminalia brownii* (leaves), *Zanthoxylum chalybeum* (leaves), *Leonotis mollissima* (leaves), *Carissa edulis* (leaves), *Tithonia diversifolia* (leaves and flowers), and *Senna didymobotrya* (leaves and pods). *In vitro* antiplasmodial activity studies of organic and water extracts were carried out against chloroquine-sensitive (D6) and chloroquine-resistance (W2) strains of *Plasmodium falciparum*. *In vivo* antiplasmodial studies were done by Peter's four-day suppression test to test for their *in vivo* antimalarial activity against *P. berghei*. Finally, cytotoxic effects and safety of the studied plant extracts were evaluated using 3-(4,5-dimethylthiazol-2-yl)-2,5-diphenyltetrazolium bromide (MTT) rapid calorimetric assay technique. The water and methanolic extracts of *T. brownii* and *S. africana* and dichloromethane extracts of *E. abyssinica*, *S. pinnata*, and *T. diversifolia* leaves revealed high *in vitro* antiplasmodial activities (IC_50_ ≤ 10 *μ*g/ml). Further, moderate *in vivo* antimalarial activities were observed for water and methanolic extracts of *L. mollissima* and *S. africana* and for dichloromethane extracts of *E. abyssinica* and *T. diversifolia* leaves. In this study, aqueous extracts of *T. brownii* and *S. africana* demonstrated high antiplasmodial activity and high selectivity indices values (SI ≥ 10) and were found to be safe. It was concluded that *T. brownii* and *S. africana* aqueous extracts were potent antiplasmodial agents. Further focused studies geared towards isolation of active constituents and determination of *in vivo* toxicities to ascertain their safety are warranted.

## 1. Introduction

Malaria is a life-threatening malady caused by a protozoan parasite transmitted via the female Anopheles mosquito's bites. It is believed to affect over 219 million people annually in 87 countries (WHO, 2016a). The most vulnerable groups affected by malarial infections entail expectant mothers and children aged between 0 and 5 years (WHO, 2016a). The World Health Organization (WHO), in the year 2017, estimated over 219 million malaria cases with over 92% of the cases occurring in sub-Saharan Africa [1].

In Kenya, 3,215,116 of *Plasmodium falciparum* were reported in the year 2017. Furthermore, the World Health Organization [1] projected that over 3.2 billion individuals residing in 91 countries are at a high risk of contracting malaria infection. In view of the rising statistics, the high mortality rates of malaria subjects are of great concern. As a result, the Global Technical Strategy for Malaria aims at reducing malaria incidences and mortalities by at least 40% before the year 2020 [1]. Many reports have indicated an increase in conventional antimalarials by the parasites [2, 3]. Moreover, associated toxicities and adverse events caused by conventional antimalarial drugs make them ineffective in malaria therapy, warranting an urgent need for alternative and complementary approaches [4]; [5].

Herbal medicine has been used throughout history and thus recognized as the oldest form of healthcare known to humanity [6]. The World Health Organization supports its use and regards it as one of the most effective strategies towards conquering the emerging diseases. It is considered one of the world's surest means of achieving total health since it is more affordable compared to conventional medicine [7]. Approximately 80% of the world's population relies on traditional medicine for their primary healthcare needs (WHO, 2015). The reliance on herbal medicine is mainly attributed to factors such as affordability, availability, and accessibility [8]. The use of medicinal plants is also integrated to most African culture and thus more acceptable than conventional medicine [9].

Increasing research data has demonstrated the potential of herbal drugs owing to the many bioactive compounds they contain [10]. These phytocompounds have been recognized as leads to some of the currently used drugs including some anticancers, analgesics, and antimalarials among others [11].

Despite the phenomenal contribution and potential of medicinal plants, especially in the treatment of malaria, there is no scientific data to validate the claimed antimalarial activities and safety. The studied medicinal plants and their parts (*Erythrina abyssinica* (stem bark), *Schkuhria pinnata* (whole plant) *Sterculia africana* (stem bark), *Terminalia brownii* (leaves), *Zanthoxylum chalybeum* (leaves), *Leonotis mollissima* (leaves), *Carissa edulis* (leaves), *Tithonia diversifolia* (leaves and flowers), and *Senna didymobotrya* (leaves and pods)), are commonly utilized by Embu community for the management of malaria [12]. Despite their longstanding usage and antimalarials, their antiplasmodial activities and toxicities are unknown, hence the current study.

## 2. Materials and Methods

### 2.1. Collection of Plants

The studied plants were collected from Embu County with the help of traditional herbalists based on their ethnomedical use and authenticated by a taxonomist at the East African Herbarium at the National Museum of Kenya, Nairobi, where voucher specimens were deposited. The collected plant materials were then air-dried for a period of one week in a well-aerated room at room temperature and then ground into a coarse powder. The respective powders were then packaged in well-labelled plastic containers and stored on dry shelves in the laboratory awaiting extraction.

### 2.2. Extraction Methods

#### 2.2.1. Aqueous Extraction

Aqueous extracts were prepared according to the method described by [13]. Briefly, 100 grams of the respective powdered plant materials was transferred into clean labelled 1000 ml capacity beakers and then 600 ml of distilled water was added. The respective concoctions in beakers were covered with aluminium foil papers and placed in a water bath (80°C) for one and a half hours to facilitate extraction. The mixtures were then filtered through Whatman's number one filter papers before subjecting them to freeze drying for 48 hrs. The dry and lyophilized samples were transferred into clean, dry, preweighed universal bottles, and their respective percentage yields were determined and stored at -20°C in a freezer until use.

#### 2.2.2. Organic Extraction

Sequential extraction using dichloromethane and methanol was adopted [13]. Briefly, 100 grams of the respective plant materials were macerated in 600 ml in 1000 ml capacity beakers at room temperature (25°C) for 48 hours. The mixtures were then filtered through double-layer Whatman's number one filter papers after which the filtrates were reduced *in vacuo* at 40°C using a rotary evaporator. Afterwards, the respective marcs were macerated in 600 ml volumes of methanol in 1000 ml capacity beakers in the same way as for dichloromethane extraction Thereafter, the menstruums were obtained by filtration and concentrated *in vacuo* using a rotary evaporator set at 56°C. The resultant extracts were transferred into clean preweighed glass bottles, and their yields were determined and stored at -20°C in a freezer until use.

### 2.3. *In Vitro* and *In Vivo* Antiplasmodial Assays

#### 2.3.1. Preparation of Stock Crude Drugs

Stock solutions of the studied crude extracts (200 *μ*g/ml) were prepared in sterile deionized water and filtered through 0.22 *μ*m membrane filters under aseptic conditions in a laminar flow hood. The water insoluble extracts were first dissolved in 0.02% dimethyl sulphoxide (DMSO) before diluting them to the required concentrations using sterile deionized water [14]. All the stock drugs were stored at -20°C and retrieved only during use.

#### 2.3.2. Culture of Malaria Parasites

Two *P. falciparum* strains (Sierra Leonean chloroquine-sensitive (D6) and Indochinese chloroquine-resistance (W2)) were obtained from the Malaria Laboratories of the Kenya Medical Research Institute (KEMRI), Nairobi, for this study. The culture medium was a variation of what was previously described by Trager and Jensen [15]. Briefly, the medium consisted of RPMI 1640 supplemented with 10% human serum, 25 mM N-2-hydroxyethylpiperazine-N-2-ethanesulfonic acid (HEPES), 25 mM NaHCO_3_, and 50 mg/ml gentamycin (0.5 ml). Human type O^+^ erythrocytes (<28 days old) served as host cells, and the cultures were incubated at 37°C in an atmosphere of 3% CO_2_, 5% O_2_, and 92% N_2_.

#### 2.3.3. *In Vitro* Antiplasmodial Assays

An *in vitro* semiautomated microdilution assay technique was used to measure the ability of the extracts to inhibit the incorporation of [G-^3^H]-hypoxanthine into the malaria parasite [16]. Aliquots of the culture medium (25 *μ*l) were added to all the wells of a 96-well flat-bottomed microculture except for row B. The test solutions (50 *μ*l) were added in triplicate to row B, and a titertek motorized hand diluter was used to make twofolds serial dilutions of each sample over a 64-fold concentration range from 200 *μ*g/ml (100%) to 3.125 *μ*g/ml (1.56%). Suspensions (200 *μ*l, 1.5% *v*/*v*) of parasitized erythrocytes (0.4% parasitemia) in the culture media were added to all rows except for row R_9_-R_12_ which contained nonparasitized erythrocytes. The plates were incubated at 37°C in an atmosphere of 3% CO_2_, 5% O_2_, and 92% N_2_. After 48 hours, each well was pulsed with 25 *μ*l of culture medium containing 0.5 *μ*Ci of [G-^3^H]-hypoxanthine and the plates were incubated for further 18 hours. The contents of each plate were harvested onto glass fiber mats, washed thoroughly with distilled water, and dried, and the radioactivity was measured using liquid scintillation.

Data from the beta counter were imported into a Microsoft Excel spreadsheet 2016, which was then transferred into an Oracle database program to determine IC_50_ values. Computation of the drug concentration causing 50% inhibition of [G-^3^H]-hypoxanthine uptake (IC_50_) was done by the interpolation of the logarithmic transformation of concentration and counts per minute (CPM) values using the following formula:

(1) $$\begin{array}{c} {\text{IC}}_{50}=\text{antilog }\left(\text{Log }X_{1}+\frac{\left(\text{Log }Y_{50}-\text{Log }Y_{1}\right)\left(\text{Log }X_{2}-\text{Log }X_{1}\right)}{\left(\text{Log }Y_{2}-\text{Log }Y_{1}\right)}\right), \end{array}$$

where *Y*_50_ is the CPM value midway between parasitized and nonparasitized control cultures, while *X*_1_, *Y*_1_, *X*_2_, and *Y*_2_ are the concentrations and CPM values for the data points above and below the CPM midpoints, respectively.

#### 2.3.4. *In Vivo* Antiplasmodial Assays

Male Swiss albino mice (6–8 weeks old, weighing 20 ± 2 g) were used as subjects. The mice were bred in standard macrolon type II cages in air-conditioned rooms at 22°C, with 50–70% relative humidity, and fed the standard diet and water *ad libitum*. *Plasmodium berghei*, a rodent malaria parasite maintained at the parasite bank of CBRD, KEMRI, was used. The assay protocol was based on Peter's 4-day suppression test [17]. The parasite strain was maintained by a serial passage of blood from an infected mouse to a naïve mouse. *P. berghei*-infected blood was obtained by cardiac puncture and parasitemia adjusted downwards to 1% by diluting it using phosphate-buffered saline with glucose (PSG; pH 7.4).

Experimental mice were randomly divided into three groups of five consisting of treatment and the controls (positive and negative) [17]. Each mouse was infected interperitoneally with 0.2 ml inoculum (1% parasitemia). The experimental group was treated with 100 mg/kg of the test sample in 0.2 ml by oral administration three hours postinfection and thereafter every 24 hours for the next three days (i.e., 24, 48, and 72 hours postinfection). The positive control was treated with 10 mg/kg of reference drug (chloroquine) while the negative control received a placebo (deionized water).

Parasitemia was determined on day four (24 hours after the last treatment) by microscopic examination of Giemsa-stained thin smears prepared from the tail blood of each mouse. Mean parasitemia in each mouse was then determined, and the difference between the mean number of parasites in the negative control group and those of the experimental groups were calculated and expressed as percentage parasitemia suppression (PS) according to the formula of [18].

(2) $$\begin{array}{c} \text{Percentage parasitemia suppression}\left(\frac{A-B}{A}\right)\times 100, \end{array}$$

where *A* is the parasitemia in the control and *B* is the parasitemia in the test group.

#### 2.3.5. In Vitro Cytotoxicity Assays

Rapid calorimetric assays by use of 3-(4,5-dimethylthiazol-2-yl)-2,5-diphenyltetra-zolium bromide (MTT) were used to determine the cytotoxicity of the extracts [13]. The assay is based on the ability of a mitochondrial dehydrogenase enzyme from viable cells to cleave the tetrazolium ring of pale yellow MTT, thus forming dark blue formazan crystals which are impermeable to the cell membrane. As a result, the formazan crystals will accumulate within healthy cells, and thus, the amount of generated formazan is directly proportional to the number of viable cells [13].

Vero (E196) cell line from the African Green Monkey's Kidney was maintained in Eagle's Minimum Essential Media (MEM) containing 10% fetal bovine serum (FBS). A cell density of 20,000 cells per well in a suspension of 100 *μ*l was seeded on 96-well plates and incubated for 24 hours at 5% CO_2_ and 37°C to achieve >90% confluence. Samples of the assay drugs and control were then added after 24 hours at a starting concentration of 1000 *μ*g/ml. Positive control (Chloroquine) was added at a concentration of 100 *μ*g/ml.

The cells were exposed to the test extracts for 48 hours after which 10 *μ*l of 10 mg/ml MTT assay reagent was added across all the wells and incubated for further 4 hours at the same condition. All the media were then removed from the plate, and 100 *μ*l DMSO was added to dissolve the formazan crystals. The plates were then read on a Multiskan EX Labsystems scanning Multiwell spectrophotometer at 562 and 690 nm as reference. The results were recorded as optical density (OD) per well at each drug concentration. The data was transferred into Microsoft Excel 2016 and expressed as the percentage of the untreated controls by the use of the formula described by [19].

(3) $$\begin{array}{c} \text{Percentage cell growth inhibition}\left(\frac{A-B}{A}\right)\times 100, \end{array}$$

where *A* is the mean OD of the untreated cells and *B* is the mean OD at each drug concentration.

The drug concentration required for 50% inhibition of cell growth was determined using nonlinear regression analysis of the dose-response curve. The selectivity index (SI) was used as the parameter for clinical significance of the test sample by comparing general toxins and selective inhibitory effect of *P. falciparum* using the equation described by [19].

(4) $$\begin{array}{c} \text{Selectivity index }\left(\text{SI}\right)=\frac{{\text{IC}}_{50}\text{ of the vero cell lines}}{{\text{IC}}_{50}\text{ of the Plasmodium cell lines}}. \end{array}$$

#### 2.3.6. Statistical Management and Data Analysis

Quantitative data was tabulated in MS Excel 2016 and exported to IBM SPSS software version 25. Data was subjected to descriptive statistics and expressed as the mean ± SEM. Thereafter, one-way analysis of variance (ANOVA) was used to determine differences between groups followed by Tukey's *post hoc* test for pairwise comparison and separation of means at *P* = 0.05. Unpaired student *t*-test statistics were performed to determine differences in longevity between the treated and untreated groups of mice.

#### 2.3.7. Ethical Clearance

Ethical clearance to conduct this research was granted by Mount Kenya University Ethics Review Committee (Ref. No. MKU/ERC/0007). The study was performed according to the set guidelines of SERU.

## 3. Results

### 3.1. Percentage Yields

Following extraction, the percentage yields of respective extracts were calculated. The results showed that, in general, high percentage yields were recorded for the aqueous leaf extracts of *Tithonia diversifolia* (11.51%) and *Senna didymobotrya* (11.14%) while *Terminalia brownii* recorded the lowest yields of 0.66%.  Table 1 presents the results.

### 3.2. *In Vitro* Antiplasmodial Assays

*In vitro* antiplasmodial results were interpreted as follows: high activity (IC_50_ ≤ 10 *μ*g/ml), moderate activity (IC_50_ of 11-50 *μ*g/ml), low activity (IC_50_ of 50-100 *μ*g/ml), and inactive (IC_50_ of ≥100 *μ*g/ml) [20], and are summarized in Table 2. In this study, out of all the aqueous and organic extracts of the studied plants that were tested against both D6 (CQ-sensitive) and W2 (CQ-resistant) strains of *P. falciparum*, 24% exhibited high activity (IC_50_ ≤ 1 0 *μ*g/ml), 43% moderate activity (IC_50_ of 11-50 *μ*g/ml), 18% low activity (IC_50_ of 50-100 *μ*g/ml), and 15% were inactive (IC_50_ ≥ 100 *μ*g/ml) (Table 2).

Aqueous extracts of *T. brownii*, and *S. africana* exhibited high antiplasmodial activity (IC_50_ ≤ 10 *μ*g/ml) while those of *E. abyssinica*, *S. pinnata*, *Z. chalybeum*, and *T. diversifolia* (leaves and flowers) exhibited moderate antiplasmodial activity (IC_50_ of 11-50 *μ*g/ml) against both strains of *P. falciparum*. Those of *C. edulis* exhibited low antiplasmodial activity (IC_50_ of 50-100 *μ*g/ml) while those of *L. mollissima* and *S. didymobotrya* leaves and pods were inactive (IC_50_ ≥ 100 *μ*g/ml) (Table 2).

Methanol extracts of *T. brownii*, *S. pinnata*, and *S. africana* exhibited high antiplasmodial activity (IC_50_ ≤ 10 *μ*g/ml) while those of *E. abyssinica, Z. chalybeum* and *T. diversifolia* (leaves and flower) exhibited moderate antiplasmodial activity (IC_50_ of 11-50 *μ*g/ml) against both strains of *P. falciparum*. Extracts of *L. mollissima*, *C. edulis*, and *S. didymobotrya* leaves exhibited low antiplasmodial activity (IC_50_ of 50-100 *μ*g/ml) while there was no activity for *S. didymobotrya* pods against the two strains of *P. falciparum* (IC_50_ > 100 *μ*g/ml) (Table 2).

Dichloromethane extracts of *E. abyssinica*, *S. pinnata*, and *T. diversifolia* (leaves) exhibited high antiplasmodial activity (IC_50_ ≤ 10 *μ*g/ml) while those of *T. brownii*, *Z. chalybeum*, *T. diversifolia* (flower), *S. didymobotrya* (leaves), and *S. africana* exhibited moderate antiplasmodial activity (IC_50_ of 11-50 *μ*g/ml) against the two strains of *P. falciparum*. However, *S. didymobotrya* (pods) and *C. edulis* exhibited low antiplasmodial activity (IC_50_ of 50-100 *μ*g/ml) while *L. mollissima* extracts were inactive (IC_50_ ≥ 100 *μ*g/ml) against the two strains of *P. falciparum* (Table 2)

There was significant difference (*P* < 0.05) in IC_50_ values between water and methanol extracts of *S. pinnata* (both strain), *E. abyssinica*, *Z. chalybeum*, and *S. africana* assayed against W2 strain of *P. falciparum*. Methanol extracts of *S. pinnata* (both strains) and *E. abyssinica* (W2) were observed to be more active than the aqueous while aqueous extracts of *Z. chalybeum* and *S. africana* assayed against W2 strain of *P. falciparum* were observed to be more active than the methanol extracts (Table 2).

There was a significant difference (*P* < 0.05) in IC_50_ values between dichloromethane and aqueous extracts of *T. brownii*, *E. abyssinica*, *S. pinnata*, *T. diversifolia* (leaves and flowers), *S. didymobotrya* (leaves and pods), *C. edulis*, and *S. africana* assayed against both strains of *P. falciparum*. Dichloromethane extracts of *E. abyssinica*, *S. pinnata*, *T. diversifolia* (leaves), *S. didymobotrya* (leaves and pods), and *C. edulis* were more active compared to the water extracts. However, aqueous extracts of *T. brownii*, *T. diversifolia* (flowers), and *S. africana* were more active than the dichloromethane extracts (Table 2).

There were significant differences (*P* < 0.05) in IC_50_ values between dichloromethane and methanol extracts of *T. brownii*, *E. abyssinica*, *S. pinnata*, *T. diversifolia* (leaves and flowers), *S. didymobotrya* (leaves and pods), *C. edulis*, and *S. africana* assayed against both strains of *P. falciparum*. Dichloromethane extracts of *E. abyssinica*, *T. diversifolia* (leaves), *S. didymobotrya* (leaves and pods), and *C. edulis* were more active compared to the methanol extracts. However, methanol extracts of *T. brownii*, *S. pinnata*, *T. diversifolia* (flowers), and *S. africana* were more active than the dichloromethane extracts.

Methanol extracts of *L. mollissima* assayed against D6 strains of *P. falciparum* were also significantly more active (*P* < 0.05) than dichloromethane extracts. The IC_50_ values of D6 strain were significantly different from those of W2 strain (*P* < 0.05) for the methanolic extracts of *T. brownii*, *S. pinnata*, *Z. chalybeum*, *T. diversifolia* (leaves and flowers), and *S. africana* (*P* < 0.05) with the extracts exhibiting higher suppression on D6 strain than on the W2 strain (Table 2).

### 3.3. *In Vivo* Antiplasmodial Assays

*In vivo* antiplasmodial results of different plant extracts assayed against *P. berghei* in mice on day four postinfection (pi) and their corresponding survival time in days are summarized in Table 3. Suppression of parasitemia was used as a measure of antiplasmodial activity and was interpreted as follows: highly active (>60%), moderately active (30-60%), lowly active (10-30%), and no activity (<10) [20]. Overall, none of the extracts exhibited high parasitemia suppression > 60%. However, 18% of the extracts exhibited moderate antiplasmodial activity and 39% exhibited low activity while 42% were inactive (Table 3).

For water extracts, *L. mollissima* and *S. africana* exhibited moderate antiplasmodial activity while *S. pinnata*, *Z. chalybeum*, *T. diversifolia* (leaves), and *C. edulis* exhibited low antiplasmodial activity. The rest of the aqueous extracts, i.e., *T. brownii*, *E. abyssinica*, *T. diversifolia* (flowers), and *S. didymobotrya* (pods and leaves) extracts, were inactive (Table 3). For methanol extracts, *S. pinnata* and *L. mollissima* exhibited moderate antiplasmodial activity while *T. brownii*, Z*. chalybeum*, *T. diversifolia* (leaves), *C. edulis*, and *S. africana* exhibited low antiplasmodial activity. The rest of the methanol extracts, i.e., *E. abyssinica*, *T. diversifolia* (flowers), and *S. didymobotrya* (pods and leaves) extracts, were inactive.

For dichloromethane extracts, *E. abyssinica* and *T. diversifolia* (leaves) exhibited moderate antiplasmodial activity while *S. pinnata* and *S. didymobotrya* (leaves and pods) exhibited low antiplasmodial activity. The rest of the extracts, i.e., Z*. chalybeum*, *T. diversifolia* (flower), and *S. africana* extracts, were inactive (Table 3).

There was a significant difference (*P* < 0.05) in parasitemia suppression for the mice treated with methanol extracts of *S. pinnata* compared to those of aqueous with the methanol extracts being more active. There was also a significant difference (*P* < 0.05) in the suppression of parasitemia in mice treated with methanol extracts of *S. pinnata*, *L. mollissima*, *T. diversifolia* (leaves), *C. edulis*, and *S. africana* compared to those of dichloromethane extracts (Table 3). Methanol extracts of *S. pinnata*, *L. mollissima*, *C. edulis*, and *S. africana* were more active compared to the dichloromethane extracts while dichloromethane extract of *T. diversifolia* (leaves) was more active compared to the water extract (Table 3).

Similarly, there was a significant difference (*P* < 0.05) in the suppression of parasitemia in mice treated with aqueous extracts of *S. pinnata*, *L. mollissima*, *C. edulis*, and *S. africana* compared to those of the dichloromethane extracts. Aqueous extracts of *L. mollissima*, *C. edulis*, and *S. africana* were more active than the dichloromethane extract while dichloromethane extract of *S. pinnata* was more active compared to the water extract (Table 3).

The mean survival time was also used as a parameter to measure efficacy of the extracts. There was a significance difference (*P* < 0.05) in the longevity (in days) between mice treated with aqueous extracts of *E. abyssinica*, *S. pinnata*, *L. mollissima*, *T. diversifolia* (leaves), and *S. africana* compared to the untreated mice. Mice treated with the aqueous extracts of *E. abyssinica*, *S. pinnata*, *L. mollissima*, and *S. africana* lived significantly longer (*P* < 0.05) than the untreated mice. However, the untreated mice lived significantly longer than those treated with aqueous extracts of *T. diversifolia* (leaves) (Table 3).

There was also a significant difference (*P* < 0.05) in the longevity (in days) between mice treated with methanol extracts of *L. mollissima*, *T. diversifolia* (leaves), and *S. africana* relative to the untreated control. Mice treated with the methanol extracts of *L. mollissima* and *S. africana* lived longer than the untreated mice. However, the untreated mice lived significantly longer (*P* < 0.05) than those treated with methanol extracts of *T. diversifolia* (leaves) (Table 3).

Additionally, there was a significant difference (*P* < 0.05) in the longevity (in days) between mice treated with dichloromethane extracts of *E. abyssinica* compared to the untreated control with the treated mice living significantly longer than the untreated mice. Mice treated with chloroquine (positive control) had a negligible level of parasitemia in the course of treatment. However, the mice showed recrudescence after treatment stopped on day four after which they all died by day 11 postinfection (Table 3).

### 3.4. *In Vitro* Cytotoxicity Assays

It is generally considered that the biological efficacy is not due to the *in vitro* cytotoxicity when the selectivity index (SI) ≥ 10 (Vonthron-Senecheau et al., 2003). In this study, the *in vitro* cytotoxicity was interpreted as follows: low selectivity index < 10 and high selectivity index > 10 according to Irungu et al. (2015). The results are summarized and are presented in Table 4. The results revealed that the aqueous extracts of *T. brownii*, *S. pinnata*, *T. diversifolia* (leaves and flowers), and *C. edulis* and methanol and dichloromethane extracts of *T. diversifolia* (flowers) as well as dichloromethane extracts of *S. africana* indicated high selectivity indices (SI) ≥ 10 against the studied strains (Table 4).

All the other extracts of *L. mollissima*, aqueous extracts of *E. abyssinica*, *Z. chalybeum* and *S. didymobotrya* (pods and leaves), and water and methanol extracts of *S. africana* as well as methanol and dichloromethane extracts of *T. brownii*, *E. abyssinica*, *S. pinnata*, *Z. chalybeum*, *T. diversifolia* (leaves and flowers), *S. didymobotrya* (pods and leaves), and *C. edulis* exhibited low selectivity indices (<10) (Table 4).

## 4. Discussion

Dichloromethane extract of *E. abyssinica* stem bark was observed to exhibit high antiplasmodial activity (IC_50_ < 10 *μ*g/ml) in this study while both water and methanol extracts were moderately active (IC_50_ of 11-50) against both chloroquine-sensitive (D6) and chloroquine-resistance (W2) strains of *P. falciparum*. However, although none of *E. abyssinica* extract was highly active *in vivo* (suppression of >60%), the dichloromethane extracts exhibited a moderate suppression of 38.85% (Table 3). These findings concur with previous studies by [21] who reported high activity for the less polar ethyl acetate extracts.

Similarly, moderate *in vivo* antiplasmodial activity has been reported for its stem bark extracts [22]. Interestingly, despite the water extract being inactive *in vivo* mice treated with these extracts lived significantly (*P* ≤ 0.05) longer than the untreated mice. This could probably be due to the presence of other therapeutic effects including anti-inflammatory, antipyretic, analgesic, and immunomodulatory properties [23, 24]

For *T. diversifolia* leaves, both water and methanol extracts exhibited moderate antiplasmodial activity (IC_50_ of 11-50 *μ*g/ml) while the dichloromethane extract exhibited high *in vitro* antiplasmodial activity (IC_50_ < 10 *μ*g/ml). High *in vitro* antiplasmodial activity was also reported for the dichloromethane extracts of the leaves collected in Rwanda [25] which coincides with the finding of this study. Similarly, the best parasitemia suppression *in vivo* (40.9%) was exhibited by the dichloromethane extract while both water and methanol only exhibited low suppression (19.0% and 20.38%, respectively). These findings concur with those reported by Oyewole et al. Additionally, prophylactic activity against Plasmodium has been reported in *T. diversifolia* [26].

Mice treated with all extracts of *T. diversifolia* leaves (including dichloromethane extracts that exhibited moderate *in vivo* suppression) significantly have shorter survival period (*P* < 0.05) than the untreated control, thus an indication of *in vivo* toxicity. Toxicity of this plant has been reported by [27] who obtained similar results.

For the flower extracts of *T. diversifolia*, moderate *in vitro* antiplasmodial activity (IC_50_ of 11-50 *μ*g/ml) was observed. However, [25] reported high *in vitro* antiplasmodial activity for the same extracts, thus the lack of concurrence with this study. This could probably be explained by the fact that the potency of the plant is influenced by factors such as geographical location, agroecologic factors, and age of the plant among other factors [28]. All these factors influence the type and concentration of phytoactive principles responsible for pharmacologic activity. *In vivo* assays revealed that all the extracts were inactive (suppression < 10 *μ*g/ml).

Consequently, there was no significant difference in longevity of the mice treated with the extracts compared to the control, thus suggesting the absence of antiplasmodial activity of this plant. It is important to note the inconsistency regarding antiplasmodial activity between the leaves and the flowers of *T. diversifolia*. This could be due to the fact that plant metabolites are not uniformly distributed in all plant parts [29].

Moderate *in vitro* antiplasmodial activity (IC_50_ values of 11-50 *μ*g/ml) was observed for all the extracts of *Z. chalybeum* leaves in this study. However, previous studies reported high *in vitro* antiplasmodial activity was reported for the leaves collected in Uganda [30]. The discrepancy is attributable to various factors such as geographical location, season, time of harvest, plant part used, and age of the plant among others [28].

*In vivo*, the plant exhibited low antiplasmodial activity (suppression of 10-30) and probably the reason why there was no significant difference in longevity of the mice treated with the extracts compared to the untreated control. However, previous studies have reported high antiplasmodial activity for the root and stem bark of this plant [25, 31–33]. This could explain the reason why roots and stem barks are more commonly used for malaria treatment.

Both methanol and dichloromethane extracts of *S. pinnata* exhibited high *in vitro* antiplasmodial activity (IC_50_ < 10 *μ*g/ml) while water extract was moderately active (IC_50_ of 11-50 *μ*g/ml). These findings support previous studies by [34] who found similar results.

For the *in vivo* assays, both water and dichloromethane extracts exhibited a low suppression (21.9% and 23%, respectively) while the methanol extract showed moderate suppression (38.31%). However, [34] reported high *in vivo* suppression (64.27%) for the water extract, thus the lack of concurrence with this study. The lack of concurrence could be explained by the fact that studies by [34] had the extract administered intraperitoneally while in this study all the extracts were orally administered to mimic the method commonly used by the herbalists. The route of drug administration influences bioavailability and the pharmacodynamics associated with therapeutic effects.

All the other extracts of *Senna didymobotrya* were inactive dichloromethane extracts of the leaves and pods that exhibited moderate (IC_50_ of 11-50 *μ*g/ml) and low (IC_50_ of 50-100 *μ*g/ml) in *in vitro* antiplasmodial activity, respectively, thus the possibility of active constituent being lipophilic in nature. Elsewhere, aqueous extracts of both pods and leaves have been reported to be inactive *in vitro*, thus the concurrence with this study [35]. Similarly, water and methanol extracts of the leaves collected in Kenya were also inactive [14], while the dichloromethane extracts of the leaves collected in South Africa were found to be moderately active *in vitro* [35]. In an *in vivo* study, it is only the dichloromethane extracts of the leaves that exhibited low suppression (14.74%), thus strengthening the suggestion of the active constituent in this plant parts being lipophilic in nature.

For *L. mollissima* leaves, both aqueous and dichloromethane extracts were inactive while methanol extracts exhibited low antiplasmodial activity. These findings are in concurrence with a previous study by [14] who also observed lack of *in vitro* activity for the water extract and low activity for the methanol extracts of the leaves collected in Kenya. However, unlike the *in vitro* assays that demonstrated lack of activity for the water extracts, moderate suppression (30-60%) was observed for the mice treated with these extracts. This evidently indicates the lack of concurrence between *in vitro* and *in vivo* assays. This phenomenon could be linked to the fact that the bioactive compounds could be prodrugs that became active upon being metabolized to active form(s) *in vivo*.

Similar findings have been reported in *Azadirachta indica* extracts [36]. Other possible reasons for the lack of concurrence between *in vivo* and *in vitro* assays could be due to the asexual erythrocytic stage of *P. falciparum* used for the *in vitro* assays, thus possibility of the plant extract being more active on other stages of the parasite and a reduction in parasitemia.

Some extracts such as aqueous extracts of *T. brownii* were inactive *in vivo* (suppression of <10 *μ*g/ml) despite them demonstrating high *in vitro* activity (IC_50_ < 10 *μ*g/ml). This could probably be due to the active constituents being biotransformed to an inactive form, poor transportation of the drug, or poor absorption of the active constituents *in vivo* [34].

In this study, all the extracts of *C. edulis* leaves were observed to exhibit low antiplasmodial activity *in vitro* (IC_50_ of 50-100 *μ*g/ml). This concurs with previous studies by [37]. Water and methanol extracts of *S. africana* were in this study observed to exhibit high *in vitro* antiplasmodial activity (IC_50_ < 10 *μ*g/ml). In *in vivo* experiments, aqueous extracts of this plant exhibited moderate activity (39.09%) while methanol extracts showed low activity (27.06%), thus confirming the use of this plant for treatment of malaria.

Furthermore, in this study, aqueous extracts of *T. brownii*, *S. pinnata*, *L. mollissima*, *T. diversifolia* (leaves and flowers), and *C. edulis* exhibited high selectivity index (SI ≥ 10), thus an indication of being safe. These observations are encouraging since the aqueous are the most commonly used for preparing herbal remedies by the traditional medical practitioners [38]. Some of the findings observed in this study corroborate those of [34] who reported a high selectivity index for the aqueous extracts of *S. pinnata*.

Additionally, aqueous extracts of *T. diversifolia* (leaves) collected from Brazil had been reported to being safe [39]. Aqueous extracts of *E. abyssinica*, *Z. chalybeum*, *S. didymobotrya* (leaves and pods), *C. edulis*, and *S. africana* were observed to exhibit low selectivity indices, an indication of cytotoxicity. Elsewhere, nitidine, the main compound responsible for antiplasmodial activity in *Z. chalybeum*, has been reported to be cytotoxic [32], thus supporting the observation in this study where all the extracts of *Z. chalybeum* exhibited low selectivity indices.

Previous studies have demonstrated the relationship between selectivity indices of drug candidates and their propensity for being potent drugs [40]. It has been shown that crude drugs with high selectivity indices and low IC_50_ values have a greater potential to providing efficacious molecules against claimed maladies [40]. Indeed, some of the studied plant extracts demonstrate better chances of providing bioactive molecules upon further evaluation against malaria.

It is worth noting that this is the first time the antiplasmodial activity of *S. africana* is being reported as to the best of our knowledge; no previous record of this is available.

## 5. Conclusions and Recommendations

### 5.1. Conclusions

Some medicinal plants used in Embu County have *in vivo* and/or *in vitro* antiplasmodial activity. However, for some plants, e.g., *Senna didymobotrya* (leaves and flowers), there was an absence of antimalarial activity, thus the need to sensitize the users against the use of these plants for malaria treatment. *In vitro* cytotoxicity was observed in the majority of plants used to treat malaria, thus the need for caution when using these medicinal plants.

### 5.2. Recommendations

#### 5.2.1. Recommendations from the Study

- Some of the studied plant extracts have *in vitro* and/or *in vivo* antiplasmodial activity
- Some of the plant extracts evaluated in this study were cytotoxic calling for caution during use

#### 5.2.2. Recommendations for Further Studies

- Specific phytocompounds responsible for the reported bioactivity in this study should be determined, isolated, and characterized
- The specific mode(s) of action for the antiplasmodial activities of some of the studied plant extracts should be elucidated
- A focused extensive toxicity study should be done to ascertain the safety of the studied plant extracts, especially those which demonstrated cytotoxicity

## Figures and Tables

**Table 1:** Percentage yields of studied plant extracts.

Plant (part used)	Voucher specimen no.	Extraction solvent	% yield
*Terminalia brownii* Fresen. (leaf)	BWW001	H_2_O	4.52
		MEOH	1.53
		DCM	0.66
*Erythrina abyssinica* Lam. Ex DC. (stem bark)	BWW002	H_2_O	5
		MEOH	2
		DCM	1.33
*Schkuhria pinnata* (Lam.) Kuntze (whole plant)	BWW003	H_2_O	9.62
		MEOH	4.66
		DCM	2.61
*Zanthoxylum chalybeum* Engl. (leaf)	BWW004	H_2_O	9.86
		MEOH	6.66
		DCM	3.33
*Leonotis mollissima* Gurke (leaf)	BWW005	H_2_O	9.87
		MEOH	6.33
		DCM	2.37
*Tithonia diversifolia* (Hemsl.) A. Gray (flower)	BWW006	H_2_O	8.66
		MEOH	5.92
		DCM	1.36
*Tithonia diversifolia* (Hemsl.) A. Gray (leaf)	BWW006	H_2_O	11.51
		MEOH	5.25
		DCM	1.34
*Senna didymobotrya* (Fresen.) Irwin & Barneby (pods)	BWW007	H_2_O	9.06
		MEOH	5.06
		DCM	1.43
*Senna didymobotrya* (Fresen.) Irwin & Barneby (leaf)	BWW007	H_2_O	11.14
		MEOH	5.75
		DCM	1.33
*Carissa edulis* (Forssk.) Vahl (leaf)	BWW008	H_2_O	7.86
		MEOH	4.54
		DCM	2.34
*Sterculia africana* (Lour.) Fiori (stem bark)	BWW009	H_2_O	8.03
		MEOH	3.45
		DCM	1.66

**Table 2:** IC_50_  values of the of the selected plant extracts assayed against CQ-sensitive (D6) and CQ-resistant (W2) strains of *P. falciparum*.

Plant (parts used)		IC_50_(*μ*g/ml)	
	Solvent of extraction	D6	W2
*Terminalia brownii* Fresen. (leaf)	H_2_O	3.34 ± 0.32^b^	5.86 ± 2.04^e^
	MEOH	4.34 ± 1.24^c^	7.09 ± 1.71^f,h^
	DCM	41.24 ± 9.78	47.31 ± 8.58
*Erythrina abyssinica* Lam. Ex DC. (stem bark)	H_2_O	47.74 ± 9.15^b^	50.11 ± 10.23^d^
	MEOH	37.37 ± 6.46^c^	34.13 ± 9.79^e^
	DCM	5.37 ± 1.59	6.99 ± 0.76^f^
*Schkuhria pinnata* (lam.) Kuntze (whole plant)	H_2_O	16.58 ± 2.08^a,b^	14.65 ± 1.09^d^
	MEOH	3.26 ± 1.25^c^	5.04 ± 0.60^e,h^
	DCM	7.28 ± 0.88	7.13 ± 0.92^f^
*Zanthoxylum chalybeum* Engl. (leaf)	H_2_O	30.97 ± 4.70	29.45 ± 8.00^d^
	MEOH	26.068 ± 7.50	39.26 ± 6.80^h^
	DCM	33.47 ± 6.78	36.25 ± 8.87
*Leonotis mollissima* Gurke. (leaf)	H_2_O	103.19 ± 8.01	107.44 ± 7.28
	MEOH	94.43 ± 8.08^c^	100.26 ± 7.3
	DCM	106.95 ± 8.71	103.76 ± 6.1
*Tithonia diversifolia* (Hemsl.) A. Gray (fruit)	H_2_O	15.28 ± 8.01^b^	16.73 ± 5.71^e^
	MEOH	12.92 ± 2.62^c^	18.14 ± 5.25^f,h^
	DCM	30.97 ± 6.54	36.12 ± 7.73
*Tithonia diversifolia* (Hemsl.) A. Gray (leaf)	H_2_O	19.81 ± 4.4^b^	22.45 ± 7.33^e^
	MEOH	18.12 ± 4.7^c^	28.18 ± 5.4^f,h^
	DCM	2.39 ± 0.92	2.0 ± 0.62
*Senna didymobotrya* (Fresen.) Irwin & Barneby (pod)	H_2_O	105.1 ± 7.53^b^	101.1 ± 3.61^e^
	MEOH	104.01 ± 6.09^c^	102.2 ± 4.9^f^
	DCM	61.02 ± 5.66	52.00 ± 10.07
*Senna didymobotrya* (Fresen.) Irwin & Barneby (leaf)	H_2_O	117.06 ± 8.87^a,b^	112.09 ± 10.1^d^
	MEOH	96.09 ± 6.46^c^	98.98 ± 8.82^e^
	DCM	23.53 ± 5.67	24.53 ± 7.0^f^
*Carissa edulis* (Forssk.) Vahl (leaf)	H_2_O	79.64 ± 17.17^b^	90.82 ± 11.34^e^
	MEOH	88.29 ± 23.76^c^	98.4 ± 6.5^f^
	DCM	53.74 ± 3.81	52.14 ± 8.56
*Sterculia africana* (Lour.) Fiori (stem bark)	H_2_O	9.32 ± 1.55^b^	9.78 ± 1.38^d,e^
	MEOH	9.73 ± 2.22^c^	10.9 ± 1.9^f,h^
	DCM	47.26 ± 5.07	41.15 ± 6.41

Values are presented as $\tilde{x}\pm \text{SD}$; ^a^*P* ≤ 0.05 MEOH (D6) vs. H_2_O (D6), ^b^*P* ≤ 0.05 DCM (D6) vs. H_2_O (D6), ^c^*P* ≤ 0.05 DCM (D6) vs. MEOH (D6), ^d^*P* ≤ 0.05 MEOH (W2) vs. H_2_O (W2), ^e^*P* ≤ 0.05 DCM (W2) vs. H_2_O (W2), ^f^*P* ≤ 0.05 DCM (W2) vs. MEOH (W2), ^g^*P* ≤ 0.05 H_2_O (D6) vs. H_2_O (W2), and ^h^*P* ≤ 0.05 MEOH (D6) vs. MEOH (W2) by one-way ANOVA with Tukey's post hoc test. DCM: dichloromethane; MEOH: methanol; H_2_O: water.

**Table 3:** Mean parasitemia, suppression, and survival time of mice in days (±SD) for extracts at 100 mg/kg.

Plant (parts used)	Solvent of extraction used	Mean parasitemia	% suppression	Mean survival time in days
*Terminalia brownii* Fresen (leaf)	H_2_O	10.8 ± 0.57	9.50 ± 3.75	8.6 ± 1.67
	MEOH	11.78 ± 0.35	11.1 ± 1.9	9.2 ± 2.04
	DCM	11.11 ± 0.48	9.88 ± 0.95	8.8 ± 1.8
*Erythrina abyssinica* Lam. Ex DC (stem bark)	H_2_O	10.87 ± 0.67	5.44 ± 2.15^b^	8.8 ± 1.8
	MEOH	11.54 ± 1.04	3.51 ± 1.6^d^	9.2 ± 2.04
	DCM	7.17 ± 0.21	36.85 ± 5.07	8.6 ± 1.67
*Schkuhria pinnata* (Lam.) Kuntze (whole plant)	H_2_O	9.32 ± 0.66	21.95 ± 7.08^b,c^	8.6 ± 1.67
	MEOH	8.18 ± 0.65	38.31 ± 6.31^d^	9.2 ± 2.04
	DCM	9.37 ± 0.18	23.88 ± 3.10	8.8 ± 1.8
*Zanthoxylum chalybeum* Engl. (leaf)	H_2_O	10.68 ± 0.33	10.60 ± 1.78	8.6 ± 1.67
	MEOH	10.6 ± 0.55	11.37 ± 3.55	9.2 ± 2.04
	DCM	10.3 ± 0.84	9.35 ± 1.23	8.8 ± 1.8
*Leonotis mollissima* Gurke (leaf)	H_2_O	7.64 ± 0.68	33.21 ± 8.08^c^	8.8 ± 1.8
	MEOH	7.63 ± 0.44	36.21 ± 5.14^d^	9.2 ± 2.04
	DCM	10.46 ± 0.82	7.97 ± 1.63	8.6 ± 1.67
*Tithonia diversifolia* (Hemsl.) A. Gray (fruit)	H_2_O	12.24 ± 0.59	7.57 ± 1.08	9 ± 2.1
	MEOH	10.76 ± 0.35	6.25 ± 2.34	9 ± 1.6
	DCM	11.62 ± 0.61	5.69 ± 1.58	8.6 ± 1.67
*Tithonia diversifolia* (Hemsl.) A. Gray (leaf)	H_2_O	10.72 ± 0.53	19.044 ± 1.6	9 ± 2.1
	MEOH	9.14 ± 0.44	20.38 ± 1.86^d^	9 ± 1.6
	DCM	7.26 ± 0.33	40.90 ± 4.81	8.6 ± 1.67
*Senna didymobotrya* (Fresen.) Irwin & Barneby (pod)	H_2_O	11.96 ± 0.63	9.89 ± 0.85	9 ± 2.1
	MEOH	10.64 ± 0.21	7.21 ± 4.22	9 ± 1.6
	DCM	11.08 ± 0.47	10.00 ± 2.69	8.6 ± 1.67
*Senna didymobotrya* (Fresen.) Irwin & Barneby (leaf)	H_2_O	12.24 ± 0.64	7.54 ± 1.72	8.6 ± 1.67
	MEOH	12.38 ± 0.45	6.48 ± 2.11	9.2 ± 2.04
	DCM	10.52 ± 0.62	14.74 ± 2.36	8.8 ± 1.8
*Carissa edulis* (Forssk.) Vahl (leaf)	H_2_O	10.33 ± 0.58	10.31 ± 4.8^c^	9.4 ± 1.68
	MEOH	9.60 ± 0.45	16.66 ± 4.34^d^	9.4 ± 1.68
	DCM	10.56 ± 0.32	6.91 ± 3.01	9.4 ± 1.68
*Sterculia africana* (Lour.) Fiori (stem bark)	H_2_O	8.40 ± 0.26	39.09 ± 5.32^d^	9.4 ± 1.68
	MEOH	7.02 ± 0.68	27.06 ± 1.6^b,c^	9.4 ± 1.68
	DCM	10.54 ± 0.32	8.50 ± 1.45	9.4 ± 1.68

^a^ *P* ≤ 0.05 between the treated and control groups for survival times. ^b^*P* ≤ 0.05 between water and methanol extracts. ^c^*P* ≤ 0.05 between water and DCM. ^d^*P* ≤ 0.05 between methanol and dichloromethane. L: leaves; SB: stem bark; WP: whole plant; F: fruit; P: pods.

**Table 4:** Selectivity indices (SI) of the selected plant part(s) assayed against CQ-sensitive (D6) and CQ-resistant (W2) strains of *P. falciparum.*

Plant (parts used)		SI	
	Solvent of extraction	D6	W2
*Terminalia brownii* Fresen. (leaf)	H_2_O	14.4	8.20
	MEOH	8.95	5.47
	DCM	3.4	2.96
*Erythrina abyssinica* Lam. Ex DC. (stem bark)	H_2_O	7.08	6.74
	MEOH	5.98	6.54
	DCM	4.6	3.53
*Schkuhria pinnata* (Lam.) Kuntze (whole plant)	H_2_O	19.78	22.38
	MEOH	4.62	2.98
	DCM	2.6	2.65
*Zanthoxylum chalybeum* Engl. (leaf)	H_2_O	0.62	0.65
	MEOH	1.01	0.67
	DCM	3.23	2.98
*Leonotis mollissima* Gurke. (leaf)	H_2_O	9.85	9.27
	MEOH	8.55	8.53
	DCM	8.48	8.17
*Tithonia diversifolia* (Hemsl.) A. Gray (fruit)	H_2_O	17.15	15.66
	MEOH	13.06	12.92
	DCM	29.81	25.55
*Tithonia diversifolia* (Hemsl.) A. Gray (leaf)	H_2_O	16.61	14.65
	MEOH	5.25	3.38
	DCM	2.20	2.62
*Senna didymobotrya* (Fresen.) Irwin & Barneby (pod)	H_2_O	2.32	2.41
	MEOH	2.44	2.48
	DCM	2.08	2.44
*Senna didymobotrya* (Fresen.) Irwin & Barneby (leaf)	H_2_O	3.04	3.17
	MEOH	3.03	2.94
	DCM	2.6	2.49
*Carissa edulis* (Forssk.) Vahl (leaf)	H_2_O	11.2	9.82
	MEOH	10.4	9.33
	DCM	9.56	9.85
*Sterculia africana* (Lour.) Fiori (stem bark)	H_2_O	7.98	7.60
	MEOH	9.65	8.61
	DCM	17.3	19.86

DCM: dichloromethane; MEOH: methanol; H_2_O: water.

## Data Availability

All the data is included within the manuscript. Any other data is available from authors upon request.
